# Active Cytomegalovirus Infection in Patients with Septic Shock

**DOI:** 10.3201/eid1210.060411

**Published:** 2006-10

**Authors:** Lutz von Müller, Anke Klemm, Manfred Weiss, Marion Schneider, Heide Suger-Wiedeck, Nilgün Durmus, Walter Hampl, Thomas Mertens

**Affiliations:** * University Hospital Ulm, Ulm, Germany

**Keywords:** sepsis, reactivation, cytomegalovirus, herpes simplex virus, diagnostic assays

## Abstract

Cytomegalovirus (CMV) is a pathogen of emerging importance for patients with septic shock. In this prospective study, 25 immunocompetent CMV-seropositive patients with septic shock and an intensive care unit stay of >7 days were monitored by using quantitative pp65-antigenemia assay, shell vial culture, and virus isolation. Within 2 weeks, active CMV infection with low-level pp65-antigenemia (median 3 positive/5 × 10^5^ leukocytes) developed in 8 (32%) patients. Infection was controlled within a few weeks (median 26 days) without use of antiviral therapy. Duration of intensive care and mechanical ventilation were significantly prolonged in patients with active CMV infection. CMV reactivation was associated with concomitant herpes simplex virus reactivation (p = 0.004). The association between active CMV infection and increased illness could open new therapeutic options for patients with septic shock. Future interventional studies are required.

## Abstract 2

Cytomegalovirus reactivation occurred in one third of patients and was associated with prolonged ventilation and stay in an intensive care unit.

Sepsis and septic shock are defined as a clinical syndrome with severe inflammatory response (*1*). Despite the availability of antimicrobial, antifungal, and supportive therapies, septic shock is fatal for about one third of patients.

Cytomegalovirus (CMV) is a human β-herpesvirus that has high seroprevalence in adults. CMV disease predominantly occurs as an opportunistic infection in patients with severe immunosuppression and rarely occurs in immunocompetent patients (*2*). Clinical diagnosis of CMV disease, without the use of virus diagnostics, is hampered by the fact that the clinical signs and symptoms are not very specific. Patients in intensive care units (ICUs) are rarely monitored for active CMV infection; therefore, the development of active CMV infection remains unrecognized in most critically ill patients.

During recent years, CMV has been discussed as an emerging pathogen in critically ill patients who are not receiving immunosuppressive therapy; however, the incidence of active CMV infection is controversial (*3**,**4*), and not all centers detected active CMV infections in these patients (*5**–**7*). Among critically ill patients, the highest incidence of active CMV infection was in patients with septic shock (*3*). The causality of sepsis, consecutive CMV reactivation, and CMV-associated pulmonary disease is supported by a mouse model of murine CMV reactivation after cecal ligation and puncture (*8**,**9*). Many factors could stimulate CMV reactivation in patients with septic shock; e.g., proinflammatory cytokines (*10**,**11*), transient immune paralysis (compensatory antiinflammatory response syndrome) (*12*), and drugs (*13*).

This pilot study investigated the incidence and the natural course of active CMV infection in patients with septic shock and different strategies for CMV monitoring. The study may stimulate future interventional trials aimed at preventing CMV-associated illness of patients with septic shock.

## Patients and Methods

### Patients

For 9 consecutive months, patients in the anesthesiologic ICU, University Hospital Ulm, Ulm, Germany, who had septic shock, were monitored for active CMV infection. We did not monitor patients who underwent splenectomy, transplantation patients, or patients receiving immunosuppressive therapy. Also, patients who had been in ICU <7 days were excluded because CMV reactivation and CMV-associated illness were expected to develop with a time delay. To define septic shock, we used the criteria established by the American College of Chest Physicians/Society of Critical Care Medicine (*14*). Clinicians were not made aware of virologic results. To avoid exogenous CMV infections, transfusions were limited to filtered leukocyte-reduced blood products. The study was approved by the local ethics committee and was in accordance with the Helsinki Declaration; informed consent was obtained.

### Virologic Examinations

CMV monitoring was performed twice during the first week of the study and once a week thereafter until the patient was discharged from ICU. Quantitative pp65-antigenemia assay; shell vial culture; and viral isolation in leukocytes, urine, and bronchial aspirate were performed as previously described (*15*). Briefly, pp65 antigenemia was determined in blood collected in EDTA tubes and subjected to dextran sedimentation (1% dextran in phosphate-buffered saline). Duplicates of 5 × 10^5^ leukocytes were placed onto glass slides, and the pp65 antigen-positive cells were evaluated by immunofluorescence assay (IFA) by using a mixture of 2 monoclonal mouse anti-pp65 antibodies (20:1; Virion, Rüschlikon, Switzerland; Argene Biosoft, Viva Diagnostika, Hürth, Germany) and goat anti-mouse immunoglobulin (Ig) G (Dianova, Hamburg, Germany) conjugated with fluorescein isothiocyanate (FITC).

Leukocytes, bronchial aspirate, and urine were investigated by shell vial culture and viral isolation with human embryonic lung fibroblasts. Three days after infection, shell vial cultures were fixed with methanol and analyzed by IFA (anti-CMV immediate early antibody, Argene Biosoft, Viva Diagnostika; FITC-conjugated goat anti-mouse IgG, Dianova). Phase contrast microscopy was used to analyze viral isolation for >6 weeks. Cytopathic effects of various viruses were confirmed by using viral typing with IFA and monoclonal antibodies.

At the initial evaluation, the following antibodies were determined semiquantitatively by using ELISA (Medac, Hamburg, Germany): CMV IgG, CMV IgM, and herpes simplex virus (HSV) IgG. Patients with antibody indices >1 were considered antibody positive.

### Clinical Data

The following values were regularly recorded: body temperature, heart rate, blood pressure, respiratory rate, need for mechanical ventilation, oxygen supply (FiO_2_), urinary output, hemodiafiltration, partial pressure of oxygen in arterial blood, pH, leukocyte count, platelet count, serum bilirubin, aspartate aminotransferase (AST), C-reactive protein, and serum creatinine. The severity of organ failure over time was recorded by monitoring the most relevant organ functions (pulmonary, cardiovascular, hematologic, hepatic) and using the Sepsis-related Organ Failure Assessment Score (SOFA) (*16*). Impairment of the central nervous system was not evaluated (Glasgow Coma Scale) because most patients received sedatives.

### Statistics

Statistical analysis was performed by using nonparametric tests (Fisher exact test, Mann-Whitney U test) and GraphPad Prism 3.02 software (GraphPad Software, San Diego, CA, USA). Significance level was set at p = 0.05.

## Results

### Patients

Among 375 patients in ICU, 38 consecutive patients with septic shock were eligible, but 13 were excluded because of CMV seronegativity (n = 5), immunosuppressive therapy (n = 2), or ICU stay <7 days (n = 6). Thus, 25 CMV-seropositive patients with septic shock and an ICU stay >7 days were enrolled in the study.

### Active CMV Infection

During the first 2 weeks after onset of septic shock, active CMV infection was detected by sensitive quantitative pp65-antigenemia assay in 8 (32%) patients (*15*). Active CMV infection was also detected by shell vial culture in 4 of these patients (in bronchial aspirate for 3 patients and in urine for 1). For 1 patient for whom shell vial culture in bronchial aspirate was positive, shell vial culture was also positive in leukocytes. Initial detection of active CMV infection was delayed when using shell vial culture (detected 1, 11, 20, and 21 days after onset of septic shock) compared with pp65-antigenemia in the same patients (0, 7, 10, and 14 days). Overall, pp65-antigenemia was low (median 3 positive/5 × 10^5^ leukocytes; range 1–17) and became nondetectable with no antiviral therapy (median 26 days after onset of active CMV infection; range 1–61 days). One patient died while CMV infection was still active.

CMV IgM antibodies were found in 2 (25%) of 8 patients with and 2 (12%) of 17 patients without active CMV infection, a difference that was not significant. Also the quantitative levels of CMV IgG and IgM antibodies did not differ between groups with and without active CMV infection (Table).

**Table:** Characteristics and clinical course of patients with septic shock, with and without active CMV infection*

		Active CMV infection	No active CMV infection	Significance
No. patients		8	17	
CMV IgG, index		12.4 (5.4–14.7)	11.7 (1.5–18.3)	NS†
CMV IgM, index		0.4 (0.28–3.94)	0.28 (0.2–1.8)	NS†
Sex, n				
	Male	5	10	NS‡
	Female	3	7	NS‡
Age, y		66 (40–78)	60 (44–78)	NS†
Primary condition, n				
	Abdominal surgery	1	7	NS‡
	Abdominal tumor	1	4	NS‡
	Pancreatitis	3	1	NS‡
	Trauma	2	5	NS‡
	Vascular surgery	1	0	NS‡
Bacteremia, n (%)		4 (50)	10 (59)	NS‡
Candidemia, n (%)		2 (25)	1 (6)	NS‡
SOFA score§		10 (7–13)	10 (7–16)	NS‡
Leukocyte count, g/L§		27 (10.4–53.3)	22.4 (7.2–74.3)	NS†
Platelet count, g/L¶		106 (37–151)	112 (37–385)	NS†
Serum creatinine, μmol/L§		183 (73–345)	160 (72–347)	NS†
Serum bilirubin, μmol/L§		27 (6–279)	54 (4–336)	NS†
Aspartate aminotransferase, U/L§		55 (7–267)	45 (9–229)	NS†
C-reactive protein, mg/L§		258 (16–456)	220 (115–437)	NS†
ICU stay after onset of septic shock, d		42 (16–87)	18 (10–42)	p = 0.0025†
Mechanical ventilation, d		39 (15–80)	16 (5–38)	p = 0.0025†
Receipt of catecholamines, d		7 (4–41)	7 (1–35)	NS†
Mortality rate, n (%)		5 (63)	6 (35)	NS‡
HSV reactivation, n (%)		6 (75)	2 (12)	p = 0.0036‡

*Median (range), unless otherwise indicated; CMV, cytomegalovirus; Ig, immunoglobulin; NS, not significant (p>0.05); SOFA, sepsis-related organ failure assessment; ICU, intensive care unit; HSV, herpes simplex virus. †According to Mann-Whitney U test. ‡According to Fisher exact test. §Highest values. ¶Lowest values.

### Characteristics of Patients with and without Active CMV Infection

Patient characteristics such as age, sex, primary disease, and severity of organ failure at time of entry into the study did not differ between patients with and without active CMV infection (Table). Hydrocortisone (200 mg/day) was given to patients in both groups; no differences between groups were noted in body temperature, leukocyte count, platelet count, serum creatinine level, serum bilirubin level, AST level, and C-reactive protein level. Systemic infection by gram-positive and gram-negative microorganisms was detected equally in both groups, and catecholamine treatment for cardiovascular dysfunction was similar for both groups.

Overall, the severity of sepsis-related failure of multiple organs, determined by SOFA score (*16*), did not differ between patients with and without active CMV infection; however, patients with active CMV infection required mechanical ventilation and ICU therapy for a longer time than did patients without active CMV infection (p = 0.0025) (Table). Although mortality rates were not significantly different between patients with and without active CMV infection (63% vs 35%; p > 0.05), the deaths occurred later (median 44 days after onset of septic shock, range 24–72 days) for patients with active CMV infection than for patients without (median 21 days, range 14–35 days) (p = 0.03).

The clinical course of patients with positive CMV shell vial culture in bronchial aspirate was associated with the longest duration of mechanical ventilation (47, 50, and 80 days) and of ICU stay (50, 71, and 87 days); however, because of the low number of cases, statistical analysis was not performed.

### Other Viral Infections

All 25 patients were HSV seropositive; viral isolation in bronchial aspirate showed reactivation of HSV in 8 (32%) patients, thereby showing for the first time that HSV and CMV reactivation were associated (p = 0.004, Table) and occurred simultaneously (Figure). Active HSV infections developed without skin or mucosal rash (occult HSV infection). Because of the low number of cases, the clinical outcome of patients with active CMV, HSV, or CMV/HSV coinfection could not be further differentiated. Viral isolation in bronchial aspirate and urine did not detect additional opportunistic viral infections such as polyoma BK virus and exogenous viral infections such as adenovirus, respiratory syncytial virus, and parainfluenzavirus in any patient.

**Figure:**
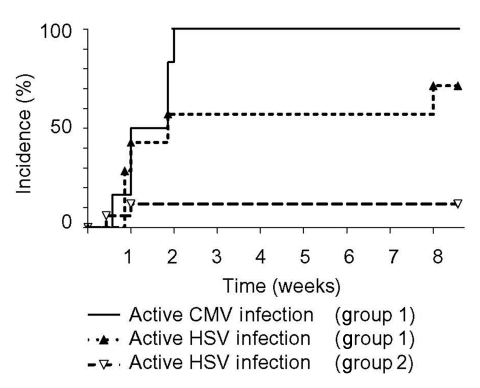
First detection of cytomegalovirus (CMV) and herpes simplex virus (HSV) reactivation after onset of septic shock. Incidence of active CMV and HSV infection is shown for patients with active CMV infection (group 1; n = 8) and without active CMV infection (group 2; n = 17). CMV reactivation occurred during the first 2 weeks after onset of septic shock (median 7 days) and was associated with HSV reactivation, which occurred during the same period. The incidence of active HSV infection was different between groups 1 and 2 (75% vs 12%; p = 0.004). Active CMV infection was detected first by CMV pp65 antigenemia; active HSV infection was detected by virus isolation of bronchial aspirates.

## Discussion

While CMV is well known as a cause of serious illness in immunosuppressed patients, it is now being discussed as a pathogen of emerging importance for patients with septic shock. Generally, active CMV infection is not recognized in such patients because critically ill patients are not routinely monitored for CMV infection.

CMV reactivation developed in one third of our patients within 2 weeks of onset of septic shock, as has been found in studies using a similar prospective study design (*3**,**11*). Diagnostic assays of different sensitivity, different patient groups, and study designs could account for discrepant results obtained by other groups (*5**,**10*). Thus, onset of active CMV infection was detected later in the retrospective studies (*4**,**17**,**18*).

Active CMV infection in patients with septic shock was characterized by a low viral load and resolved within a few weeks without antiviral therapy. We hypothesize that upon CMV reactivation, patients with septic shock could mount a protective antiviral immune response, which was different from the immune response of most patients after transplantations (*19*); however, this hypothesis remains to be confirmed.

In a previous study we compared different assays for CMV monitoring of patients with organ transplants and demonstrated equal sensitivity of our pp65 antigenemia assay and CMV PCR of blood cells but lower sensitivity of shell vial culture, CMV PCR in plasma, and CMV mRNA detection by nucleic acid sequence-based amplification (*15*). Because of low viral loads, the incidence of active CMV infection could be easily underestimated by less sensitive assays for patients with septic shock, which was shown here in that shell vial culture in blood cells detected only 1 patient with active CMV infection. Less sensitive assays could have been also the problem of studies that failed to detect active CMV infection in critically ill patients (*5**–**7*). We assume that assays with sensitivity similar to that of our pp65-antigenemia assay (e.g., CMV PCR of blood cells) may be equally used for CMV monitoring of patients with septic shock, considering the results of patients who had received transplants (*3**,**11**,**15*).

Shell vial culture was more likely to detect active CMV infection in bronchial aspirate than in urine or blood cells. Pulmonary CMV infection may be relevant for patients with septic shock (*8**,**20*). Shell vial culture of urine was rarely positive for CMV in patients with septic shock, a finding which differed for patients having received a kidney transplant (*21*).

As expected, quantitative analysis of CMV IgG and IgM antibodies could not discriminate between patients with and without active CMV infection. CMV IgG antibodies were analyzed to identify patients with previous CMV infection (CMV-seropositive patients); however, diagnosis of active CMV infection by detection of CMV IgM antibodies or rising CMV antibody titers are no longer recommended when sensitive CMV monitoring by pp65-antigenemia assay or CMV PCR are available because the information they provide is limited.

The clinical role of active CMV infection in patients with septic shock is an area of ongoing discussion (*4*). We demonstrated that active CMV infection is associated with prolonged ventilation time and ICU stay. Ventilation time and ICU stay were more prolonged in a subgroup of patients for whom shell vial culture in bronchial aspirate was positive. CMV infection was associated with pulmonary disease despite low pp65 antigenemia and self-limiting CMV infection. We suppose that immunopathologic mechanisms could contribute to CMV-associated illness (*22*) in addition to direct cytopathic effects of the infection (*20*). Association of tumor necrosis factor and pulmonary immunopathologic features of active CMV infection was recently confirmed in a mouse model showing murine CMV reactivation after cecal ligation and puncture (*9*).

Deaths occurred later for patients with active CMV infection than for those without active CMV infection. This finding could be the consequence of CMV-associated disease, as has been suggested (*17*). Although our study was not designed to clarify the causality between active CMV infection and increased illness, we argue that active CMV infection increases illness and not vice versa. In the mouse model of CMV reactivation, the causality between sepsis, CMV reactivation, and pulmonary disease has already been shown (*9*).

Recently, reactivations of HSV and human herpesvirus 6 have been reported in critically ill patients (*7**,**23*). We demonstrated for the first time an association between active HSV and CMV infection (p = 0.004). HSV was isolated from bronchial aspirate in the absence of skin and mucosal lesions, whereas other herpesviruses, such as varicella-zoster virus, could not be isolated. The coincidence of HSV and CMV reactivation during the first 2 weeks of septic shock suggests a common trigger mechanism for herpesvirus reactivations. In future studies, more sensitive assays (e.g., PCR) may be used to analyze the incidence of other occult herpesvirus reactivations. Reactivation of polyoma BK virus, which commonly causes opportunistic infection after transplantation, was not detected by virus isolation. This finding leads to the hypothesis that the conditions that stimulate polyomavirus reactivation and those that stimulate CMV and HSV reactivation may differ. The absence of exogenous viral infection (e.g., adenovirus, respiratory syncytial virus, parainfluenzavirus) strengthens the suggestion that exogenous nosocomial viral infections are uncommon in patients in ICUs (*24*). Thus, monitoring for viral infections could focus on endogenous herpesvirus reactivations in patients with septic shock. Immunosuppression and proinflammatory cytokines, drugs, or combinations are presumed to be involved in herpesvirus reactivations; however, the exact mechanisms are still elusive for patients with septic shock (*13**,**25*).

After organ transplants, CMV-associated illness and death could be reduced by early antiviral therapy; however, delayed therapy has been less effective (*2*). Anecdotal reports show that critically ill patients with already established CMV organ disease may not benefit from antiviral therapy (*3**,**4**,**20*). The effect of preemptive antiviral therapy or antiviral prophylaxis has not been tested so far in patients with septic shock; however, in the mouse model, prophylactic treatment with ganciclovir prevented murine CMV reactivation and CMV-associated pulmonary fibrosis (*9*).

Despite the low patient number in this and previous studies, we suggest that CMV is a pathogen of emerging importance that can no longer be ignored for patients with septic shock. Thus, interventional studies aimed at preventing CMV-associated illness in patients with septic shock are needed.
